# Radar Position Estimation by Sequential Irradiation of ESM Receivers

**DOI:** 10.3390/s21134430

**Published:** 2021-06-28

**Authors:** Petr Hubáček, Jiří Veselý, Jana Olivová

**Affiliations:** Department of Communication Technologies, Electronic Warfare and Radars, University of Defence in Brno, 66210 Brno, Czech Republic; petr.hubacek@unob.cz (P.H.); jiri.vesely@unob.cz (J.V.)

**Keywords:** localization method, positioning technique, covariance matrix, target localization

## Abstract

In this article, a new technique for determination of 2D signal source (target) position is proposed. This novel approach, called the Inscribed Angle (InA), is based on measuring the time difference of sequential irradiation by the main beam of the target antenna’s radiation pattern, using Electronic Support Measures (ESM) receivers, assuming that the target antenna is rotating and that its angular velocity is constant. In addition, it is also assumed that the localization system operates in a LOS (Line of Sight) situation and that three time-synchronized sensors are placed arbitrarily across the area. The main contribution of the article is a complete description of the proposed localization method. That is, this paper demonstrates a geometric representation and an InA localization technique model. Analysis of the method’s accuracy is also demonstrated. The time of irradiation of the receiving station corresponds to the direction in which the maximum received signal strength (RSS) was measured. In order to achieve a certain degree of accuracy of the proposed positioning technique, a method was derived to increase the accuracy of the irradiation time estimation. Finally, extensive simulation was conducted to demonstrate the performance and accuracy of our positioning method.

## 1. Introduction

Localization of non-cooperative signal emitters (targets) is a common task in many security and surveillance systems. These systems can be applied to a wide variety of areas, such as military applications [1,2], target tracking, electronic intelligence systems [3], perimeter protection systems [4], or location-based services [5]. The most commonly used approaches, or techniques, for measuring a target position are Time of Arrival (TOA) [6], Time Difference of Arrival (TDOA) [7,8], Received Signal Strength (RSS) [9], Doppler Difference (DD) [10], Angle of Arrival (AOA) [11], and combinations of these techniques [12,13]. The localization techniques can be described by a number of different features, such as accuracy, complexity, ambiguity, cost, etc.

In this paper, we focus on the time-based localization systems. Their general operation principle is based on either measuring the Time Difference of Arrival or measuring the Time of Arrival of the signal of interest to different receiver positions. These systems allow instantaneous target position measurement but only if the condition of simultaneous irradiation of all the ESM receivers [14] of the localization system is satisfied. However, localization of Low Probability of Intercept (LPI) targets [15,16], especially in military applications, can be problematic in these systems. One of the features of the LPI signal emitters (radars) is a side lobes suppression of their antenna patterns. This means that the condition of simultaneous irradiation of the time-based localization system may not be satisfied and that the target cannot be localized. There are several approaches for overcoming this limitation. A baseline shortening of the time-based positioning systems is one of the possible approaches [17]. Although the short baseline significantly increases the probability of the system simultaneous irradiation, it also reduces the accuracy of the target localization, assuming a constant TOA or TDOA measurement error [18]. The second group of possible approaches is based on a certain degree of cooperation between the target and the positioning system sensors. This group includes, for example, the sequential TDOA method [19]. However, this cooperation can be a major limitation, especially for military applications.

All the recent time-based localization systems are able to measure both the TOA and the amplitude of the target signal. We propose a new localization technique in 2D that is based on measuring the time difference of sequential irradiation of three receiving localization system stations by the main beam of the target antenna radiation pattern. If we further assume that the angular velocity of the target antenna rotation is constant and that it is known (the angular velocity is measurable as well as the time difference of station irradiation), we are able to determine two inscribed angles [20] in two circles. The position of the target is then determined by the intersection of these two circles. As the knowledge of the inscribed angle is the key parameter of the proposed method, this localization technique is called Inscribed Angle method. In connection with the assumption of the constant angular velocity of the target antenna rotation, a typical application of the proposed method can be the tracking of fishing boats equipped with small maritime radar because these radars are just typical of constant antenna rotation speed and circular scanning of the area. Another possible application of the InA method is the localization of long-range surveillance radars where only irradiation by radar antenna main beam (without antenna sidelobes) is available.

This paper is organized as follows: Section 2 introduces a geometric representation of the InA method and shows mathematical model of this method and its algorithm, including a demonstration of the method performance. Section 3 briefly describes accuracy analysis of the proposed method. Section 4 presents simulation results demonstrating the accuracy of our positioning method. Section 5 describes the technique for increasing accuracy of the irradiation time estimation.

## 2. Geometric Representation and Description of the InA Localization Technique Model

In this section, a typical scenario for using the InA localization method is described. Furthermore, a mathematical model of the method and its final proposed solution are also presented.

**Figure 1:**
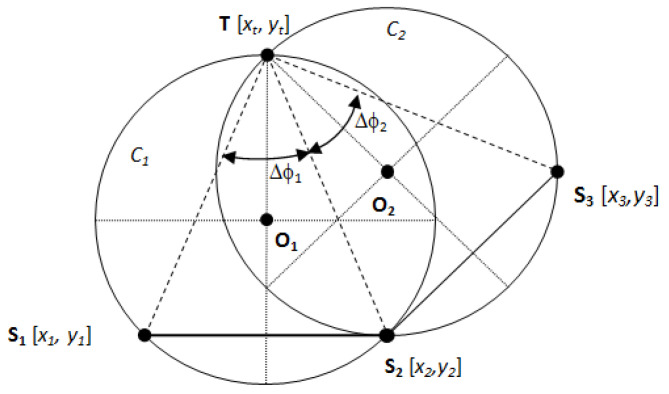
Sensor network and target arrangement.

A network, composed of three fixed positions and time-synchronized receiving sensors, is assumed. In this example, there are sensors **S**_1_, **S**_2_, **S**_3_, and one target, **T**. The number of sensors *N* = 3 represents a sufficiently determined system in 2D. The arrangement of such a network is shown in Figure 1. It is assumed that each sensor is able to measure irradiation time *T_Ri_*, i.e., the time when the sensor *i* is irradiated by the main beam of the target antenna radiation pattern. The times of irradiation can be, for clarity, written in the form of vector **T**_R_ = [*T_R_*_1_, *T_R_*_2_, *T_R_*_3_]. Next, it is assumed that the target antenna is rotating and that its angular velocity *Ω* is constant. It means that an arbitrary sensor can estimate the rotation period of the target antenna *T_A_* from two consecutive times of irradiation.

If the coordinates of the target and sensors are **T** = [*x_t_*, *y_t_*], **S**_1_ = [*x*_1_, *y*_1_], **S**_2_ = [*x*_2_, *y*_2_] and **S**_3_ = [*x*_3_, *y*_3_], then the time of irradiation *T_Ri_* of the sensor *i* is

(1) $$T_{Ri}=T_{0}+\frac{\alpha_{i}}{\Omega }$$

where *T*_0_ is a priori unknown time when main lobe of the transmitter antenna pattern is directed to *x* axis. This orientation (as a mathematical zero angle) is used for simplicity instead of standard azimuth angle oriented northward and clockwise, and *α_i_* expresses the direction to the target from *i* sensor at the moment of its irradiation by the main antenna beam of the signal source.

(2) $$a_{i}=\tan^{-1}\left(\frac{y_{t}-y_{i}}{x_{t}-x_{i}}\right)$$

This set of Equations (1) and (2) represents the model of the InA localization method.

Using this method model, the target position can be found as follows: First, if the irradiation times and the period of target antenna rotation are known (are measured), then the angular velocity of the target antenna *Ω* can be determined as

(3) $$\Omega =\frac{2\pi }{T_{A}}$$

Next, the inscribed angles in circles *C*_1_ and *C*_2_ can be computed as

(4) $${\Delta \Phi }_{1}=\left(T_{R2}-T_{R1}\right).\Omega$$

(5) $${\Delta \Phi }_{2}=\left(T_{R3}-T_{R2}\right).\Omega$$

If the inscribed angles are determined, it is possible to describe the circles *C*_1_ and *C*_2_, i.e., to find their center coordinates **O_1_**, **O_2_** and their radii *r*_1_, *r*_2_.

For circle *C*_1,_ it can be done as follows: In order to simplify determination of the circle *C*_1_ parameters, it is advisable to transform the sensor arrangement from a standard coordinate system {*x*, *y*} into a new coordinate system {x*, y*} by translation and rotation via the following transformation equations:

(6) $$x^{*}=\left(x-x_{1}\right).\cos\left(\beta_{1}\right)+\left(y-y_{1}\right).\sin\left(\beta_{1}\right)$$

(7) $$y^{*}=-\left(x-x_{1}\right).\sin\left(\beta_{1}\right)+\left(y-y_{1}\right).\cos\left(\beta_{1}\right)$$

where $\beta_{1}=\tan^{-1}\left(\frac{y_{2}-y_{1}}{x_{2}-x_{1}}\right)$.

Then, the new sensor coordinates are $S_{1}^{*}\;\left[0,\;0\right]$, $S_{2}^{*}\;\left[a,\;0\right]$ and the center of the circle *C_1_* has coordinates $O_{1}^{*}\;\left[x_{o1}^{*},\;y_{o2}^{*}\right]$, where

(8) $$x_{o1}^{*}=\frac{a}{2}=\frac{\sqrt{{\left(x_{2}-x_{1}\right)}^{2}+{\left(y_{2}-y_{1}\right)}^{2}}}{2}$$

(9) $$y_{o1}^{*}=\cot\left({\Delta \Phi }_{1}\right).x_{o1}^{*}$$

The radius of *C*_1_ is

(10) $$r_{1}=\sqrt{x_{o1}^{*2}+y_{o1}^{*2}}$$

A transformation of the *C*_1_ circle center back into the standard coordinate system {*x, y*} is provided by the following formulas:

(11) $$x=x^{*}.\cos\left(\beta_{1}\right)-y^{*}.\sin\left(\beta_{1}\right)+x_{1}$$

(12) $$y=x^{*}.\sin\left(\beta_{1}\right)+y^{*}.\cos\left(\beta_{1}\right)+y_{1}$$

Similarly, the center $O_{2}\;\left[x_{o2},\;y_{o2}\right]\;$ of the circle *C*_2_ and its radius *r*_2_ can be determined. Note that in this case, the sensor coordinates are $S_{2}^{*}\;\left[0,\;0\right]$ and $S_{3}^{*}\;\left[b,\;0\right]$ in the new coordinate system and the angle $\beta_{2}=\tan^{-1}\left(\frac{y_{3}-y_{2}}{x_{3}-x_{3}}\right)$ is used in transformation Equations (6), (7), (11), and (12).

Finally, the target position can be found as an intersection of the circles *C*_1_ and *C*_2_. If the equations of circles are expressed as

(13) $${\left(x-x_{o1}\right)}^{2}+{\left(y-y_{o1}\right)}^{2}=r_{1}^{2}$$

(14) $${\left(x-x_{o1}\right)}^{2}+{\left(y-y_{o1}\right)}^{2}=r_{1}^{2}$$

then a new system of these equations is obtained and is equivalent to (13), (14) after the transformation of $O_{1}\;\left[x_{o1},\;y_{o1}\right]\to O_{1}^{*}\left[0,0\right],\;O_{2}\;\left[x_{o2},\;y_{o2}\right]\to O_{2}^{*}\left[c,0\right]$:

(15) $$x^{*2}+y^{*2}=r_{1}^{2}$$

(16) $${\left(x^{*}-c\right)}^{2}+y^{*2}=r_{2}^{2}$$

where *c*$=\sqrt{{\left(x_{o2}-x_{o1}\right)}^{2}+{\left(y_{o2}-y_{o1}\right)}^{2}}$.

By subtracting Equation (16) from Equation (15), we get the following formula:

(17) $$2.c.x^{*}-c^{2}=r_{1}^{2}-r_{2}^{2}$$

By rearranging Equation (17), the formula for the $x_{t}^{*}$ target coordinate is:

(18) $$x^{*}=x_{t}^{*}=\frac{c^{2}+r_{1}^{2}-r_{2}^{2}}{2.c}$$

Using (15), the formula for the $y_{t}^{*}$ target coordinate is:

(19) $$y_{1,2}^{*}=y_{t1,2}^{*}=\pm \sqrt{r_{1}^{2}-x_{t}^{*2}}$$

Equation (19) shows that the proposed method provides two target positions. Thus, the InA localization technique is ambiguous. However, it is clear that one of the target positions is always at the position of sensor that lies on both circles (it is from Figure 1). Then the question of unambiguity need not be further solved in practical applications. A transformation of the target position back into the standard coordinate system {*x, y*} is provided by the following formulas:

(20) $$x_{t}=x_{t}^{*}.\cos\left(\gamma \right)-y_{t}^{*}.\sin\left(\gamma \right)+x_{o1}$$

(21) $$y_{t}=x_{t}^{*}.\sin\left(\gamma \right)+y_{t}^{*}.\cos\left(\gamma \right)+y_{o1}$$

where $\gamma =\tan^{-1}\left(\frac{y_{o2}-y_{o1}}{x_{o2}-x_{o1}}\right)$.

From a practical point of view, the derived algorithm of the InA method is implemented in the following way. Firstly, the *T_R_*_1_ to *T_R_*_3_ are measured and *T_A_* is derived from them. Secondly, the parameters ΔΦ_1_ and ΔΦ_2_ are determined. Then, all the parameters of the circles *C*_1_ and *C*_2_ are computed. Finally, the target coordinates *x_t_*, *y_t_* are found as the intersection of the circles.

The following presented simulation shows the InA method performance. A network was assumed, composed of three receiving sensors with coordinates **S**_1_ [−10 km, 5 km], **S**_2_ [0, 0], and **S**_3_ [10 km, 5 km]. The target position was **T** [−5 km, 50 km], and it irradiated the sensor system twice. The vectors of the measured *T_Ri_* were

$$T_{R}^{1}=\left[1.0972\;s,\;1.1976\;s,\;1.3036\;s\right]\;\;(\mathrm{the}\;\mathrm{first}\;\mathrm{irradiation})\;\mathrm{and}\;T_{R}^{2}=\left[4.0972\;s,4.1976\;s,\;4.3036\;s\right]\;\;(\mathrm{the}\;\sec\mathrm{ond}\;\mathrm{irradiation}).$$

**Figure 2:**
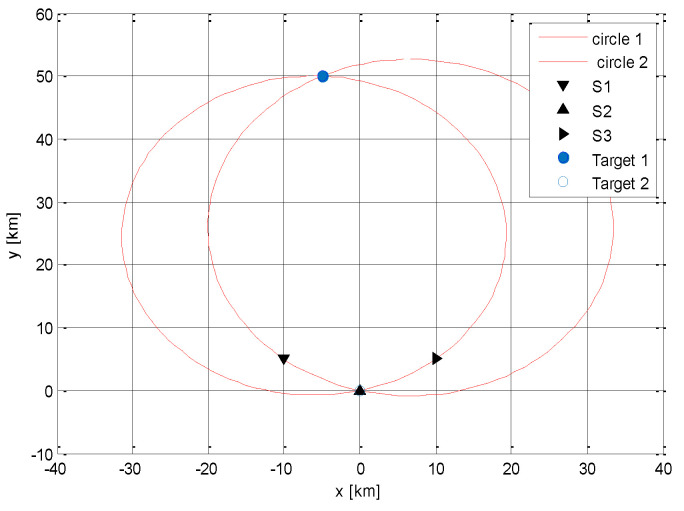
Example of the InA method performance.

Then, the estimation of the rotation period of the target antenna was *T_A_* = 3 s and appropriate inscribed angles were ΔΦ_1_ = 12.51 deg and ΔΦ_2_ = 12.72 deg. Finally, the calculated target positions were **T_1_** = [−5 km, 50 km] and **T_2_** = [0, 0]. Figure 2 shows a graphical interpretation of this simulation.

The achieved simulation results show that the proposed method works correctly. It is also important to note that in the presence of multiple target signals, it is necessary to perform thorough deinterleaving, i.e., to separate the signals of individual targets before applying the proposed algorithm. This can be done, for example, using knowledge (or measurement) of the parameters of the signal of individual targets. However, the solution to the problem of deinterleaving performance is not the subject of the present paper.

## 3. Accuracy Analysis of the InA Localization Method

In estimation theory, the Cramér–Rao Lower Bound (CRLB) expresses a lower bound on the variance of unbiased estimators of a deterministic parameter [21]. The approach based on CRLB theory is used for analyzing the accuracy of the InA method in the presence of noise. According to [22,23,24], it is possible to determine the covariance matrix $C\left(T\right)$ of the InA method as

(22) $$CRLB\left(T\right)=C\left(T\right)=J\left(\hat{T_{R}}\right).C_{p}\left(\hat{T_{R}}\right).J{\left(\hat{T_{R}}\right)}^{T}$$

where $J\left(\hat{T_{R}}\right)\;$ is Jacobian Matrix. It consists of partial derivatives of the function $f\left(T_{R},S_{1..3}\right),\;$ with respect to the variables *T_R1_* to *T_R3_* at the point $\hat{T_{R}}$. The $\hat{T_{R}}$ is the vector of the measured times of irradiation, i.e.,$\;\hat{T_{R}}$ is an estimation of **T_R_**,

(23) $$J\left(\hat{T_{R}}\right)=\left[\frac{\partial f\left(\hat{T_{R}},S_{1..3}\right)}{\partial T_{R}}\right]=\left[\begin{array}{ccc} \frac{\partial x\left(\hat{T_{R}},\;S_{1..3}\right)}{\partial T_{R1}} & \frac{\partial x\left(\hat{T_{R}},\;S_{1..3}\right)}{\partial T_{R2}} & \frac{\partial x\left(\hat{T_{R}},\;S_{1..3}\right)}{\partial T_{R3}}\\ \frac{\partial y\left(\hat{T_{R}},\;S_{1..3}\right)}{\partial T_{R1}} & \frac{\partial y\left(\hat{T_{R}},\;S_{1..3}\right)}{\partial T_{R2}} & \frac{\partial y\left(\hat{T_{R}},\;S_{1..3}\right)}{\partial T_{R3}} \end{array}\right]$$

and $C_{p}\left(\hat{T_{R}}\right)$ is the covariance matrix of the vector $\hat{T_{R}}$. If the times of irradiation of particular sensors are measured independently, as in the proposed method, the matrix $C_{p}\left(\hat{T_{R}}\right)$ becomes a diagonal matrix in the following form:

(24) $$C_{p}\left(\hat{T_{R}}\right)=\left[\begin{array}{ccc} \sigma_{TR1}^{2} & 0 & 0\\ 0 & \sigma_{TR2}^{2} & 0\\ 0 & 0 & \sigma_{TR3}^{2} \end{array}\right]$$

where $\sigma_{TRi}^{2}$ is a dispersion of the *T_Ri_* parameter. An example of finding partial derivatives of the function $f\left(T_{R},S_{1..3}\right)$ is shown in Appendix A.

The defined covariance matrix $C\left(T\right)\;$ represents a confidence region including the “true” target position with a certain probability level [25]. From a physical point of view, the covariance matrix expresses an error ellipse with a certain probability of the “true” target occurrence [26]. The computation of error ellipse parameters, i.e., the length of axes and their directions, is described in detail in [27].

## 4. Accuracy Evaluation of the InA Localization Technique

**Figure 3:**
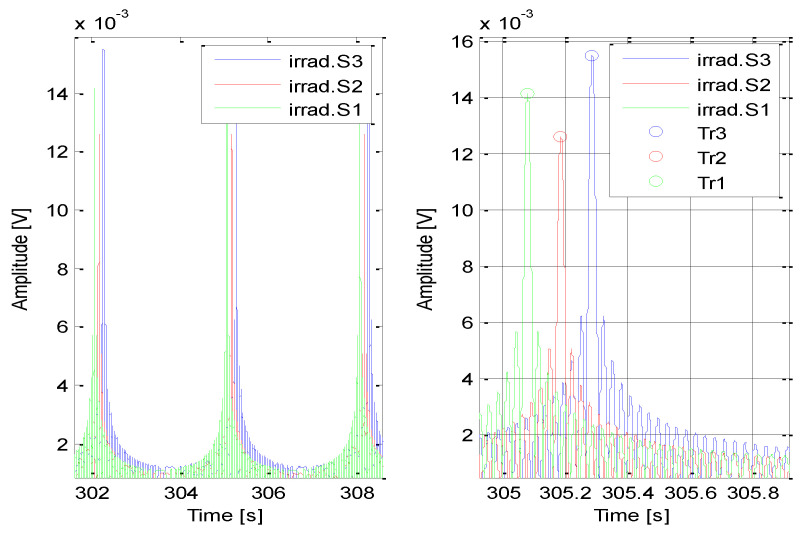
Examples of sensor irradiations.

Mathematical background for describing the accuracy of the InA method was provided in the previous section. This section presents the simulated performance results of the proposed technique in terms of its accuracy. The same sensor arrangement was assumed as in the previous example (see Section 2). Then, it was also assumed that the *T_Ri_* were measured at each sensor independently with a standard deviation equal to 0.1 ms and that all the sensors were time-synchronized. The antenna of the fixed target rotated with period *T_A_* = 3 s, and the simulation was so long that the main beam of the target antenna was able to irradiate the sensors several times. An example of such irradiation for particular sensors is shown in Figure 3.

**Figure 4:**
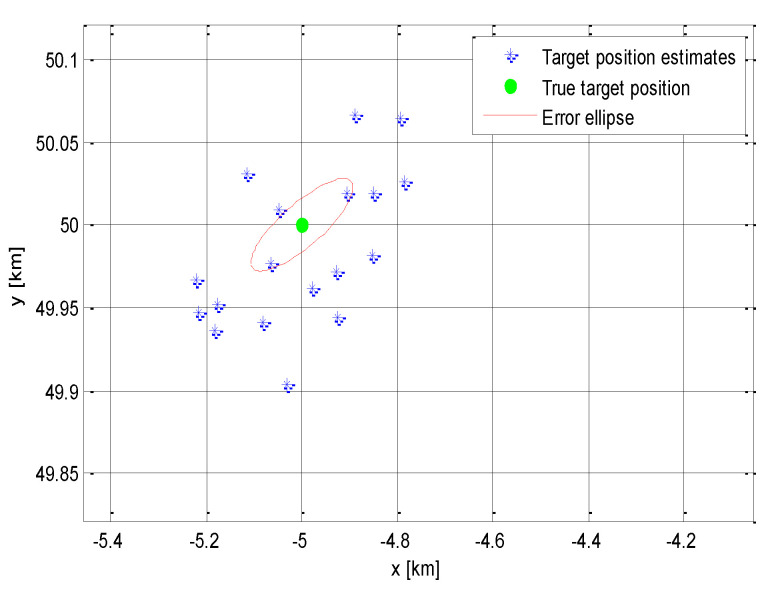
Measured target positions and error ellipse.

In this presented simulation, the system was irradiated 20 times. That means it was possible to extract *N* = 20 vectors of $\hat{T_{R}}$ and then calculate *N* estimates of the target position $\hat{T}$. The measured target positions are shown in Figure 4.

This corresponds to the mean estimated position of the target ${\hat{T}}_{mean}$ [−5.05 km, 50.005 km]. The accuracy of the target localization can be quantified by the following covariance matrix:

$$C\left(T\right)=\left[\begin{array}{cc} 0.0455 & 0.0096\\ 0.0096 & 0.0031 \end{array}\right]$$

The error ellipse axes lengths are *A_e_* = 109.1 m and *B_e_* = 16.0 m.

From the achieved results, it is clear that the proposed method had a significantly larger cross-range error than the range one. In addition, it is also clear that the target position error appeared to be strongly dependent on the standard deviation of the irradiation time measurement $\sigma_{TRi}$. This dependence could be generally assessed using the error ellipse axes length or the CEP (Circular Error Probability) parameter. For simplicity, it is also possible to use the range deviation parameter *R_mean_* that is introduced as

(25) $$\Delta R_{mean}=\frac{\sum_{i=1}^{N}\hat{T_{i}}-T}{N}$$

where ${\hat{T}}_{i}$ is the *i* measurement of the target position, and *N* is the overall number of these measurements. Note that Δ*R_mean_* = 175.5 m in the above presented simulation.

**Figure 5:**
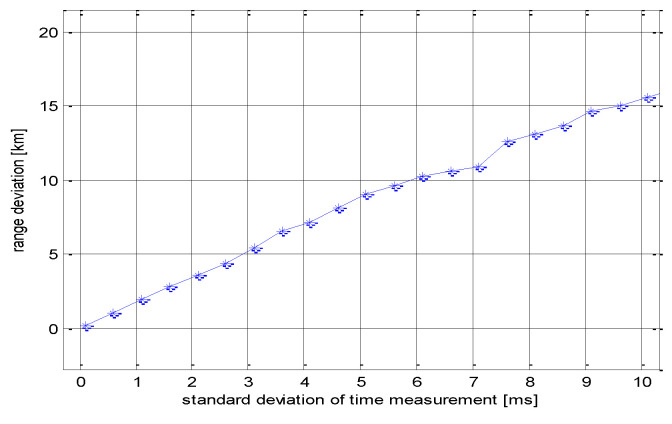
Range deviation of the target.

Thus, another simulation was performed, and the same sensor system configuration and target position were assumed as in the previous examples. The mean range deviation Δ*R_mean_* was then calculated for interval of $\sigma_{TRi}$ from 0.1 ms to 10 ms. *N* = 100 target position measurements were considered for each $\sigma_{TRi}$. The results of this test are shown in Figure 5.

The graph shows that the accuracy of the target location decreased sharply as the measurement error of *T_R_i* increased. From a “practical application of this presented method” point of view, the acceptable value of $\sigma_{TRi}$ was in the interval from 0.1 ms to 1 ms. The above simulation also makes it possible to determine the effect of sensor placement. It turns out that the application of the proposed method for sensor arrangement with distances of meters between particular sensors is problematic due to the requirement for accuracy of irradiation time measurement. In this case, the difference of irradiation times of individual sensors will be too short and thus the requirement for its measurement accuracy will increase rapidly. Thus, in principle, the method will work correctly, but its technological implementation will be very difficult. For short-range targets, influencing the transmitting platform inherent rotation and tilt effect will be a problem.

Hence, these results represent a very strict requirement for the technological solution of the ESM receiver. Therefore, the next section presents one of the possible solutions to overcome this limitation.

## 5. The Technique to Increase Accuracy of Irradiation Time Estimation

**Figure 6:**
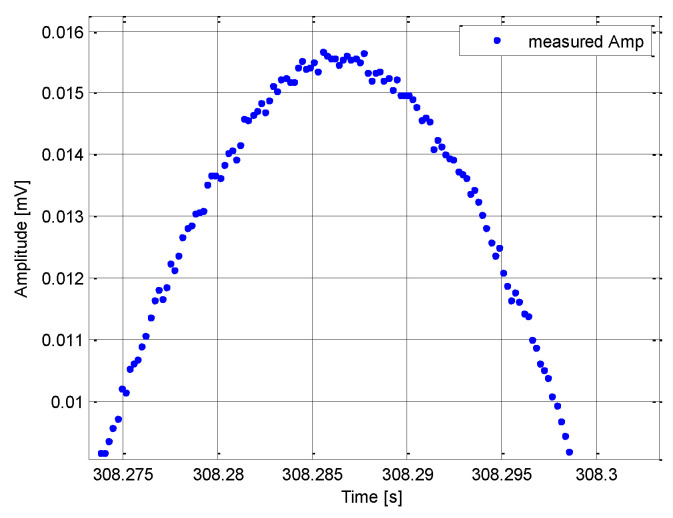
Measured amplitude of the received signal.

Determination of the time of irradiation is generally based on a continuous measurement of the received signal amplitude by the ESM receiver, followed by finding the maximum amplitude for which the *T_R_* is extracted. The accuracy of determining the *T_R_* in the presence of noise depends on both the signal-to-noise ratio (SNR) of the received signal and the parameters of the ESM receiver [28,29]. The correct extraction of the time of irradiation is significantly affected by the flat shape of the target antenna main beam. A graph of the measured amplitude of the received signal in the presence of noise is shown in Figure 6.

**Figure 7:**
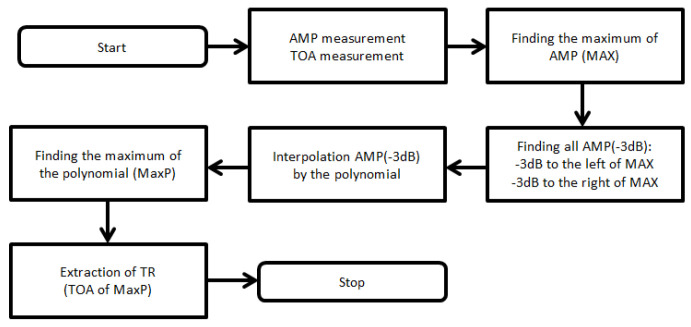
Algorithm to increase accuracy of the *T_R_* extraction.

The accuracy analysis of the InA method showed that it is necessary to ensure the most accurate measurement of the times of irradiation for the correct operation of the InA method. Therefore, the following algorithm was proposed to improve the *T_R_* extraction. The core of the algorithm is based on interpolation of the measured amplitude of the received signal by a polynomial and subsequently finding its local maximum [30]. The irradiation time then corresponds to this local maximum. Figure 7 shows the flowchart of the proposed algorithm.

**Figure 8:**
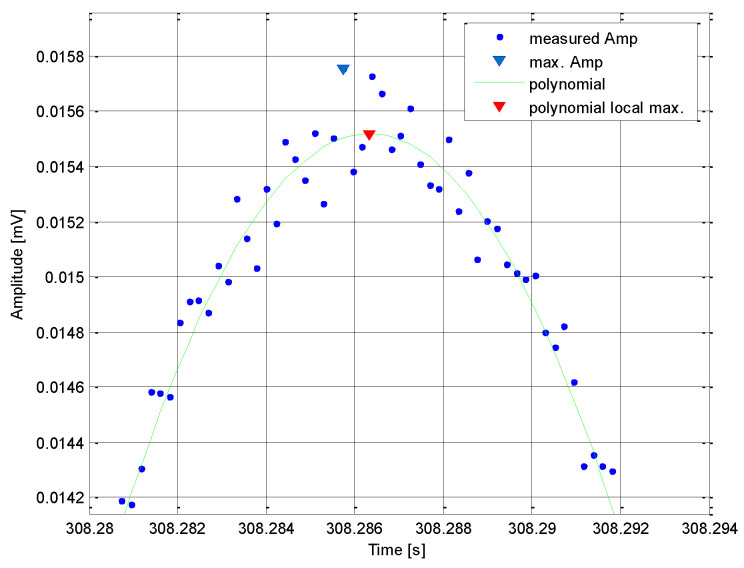
Simulation of the algorithm to increase accuracy of the *T_R_* extraction.

Performance of this algorithm was tested by a simulation with the following parameters: The target antenna had its main beam width *θ_-_*_3*dB*_ = 0.5 deg and rotated with the period *T_A_* = 3 s. The pulse repetition interval of a pulse radar signal was set to *PRI* = 100 s, and the *SNR* of the signal was approximately 12 dB. Thus, the receiver was irradiated with *M* = 40 pulses, as shown in Figure 8.

Without the use of the proposed algorithm, the time of irradiation *T_R_* would have been extracted for the maximum measured amplitude *A_max_*. However, when these *M* measured amplitudes were interpolated by a polynomial of degree 4, then the *T_R_* could be extracted at a point more closely corresponding to the maximum receiver irradiation by the main target antenna beam.

## 6. Conclusions

The presented Inscribed Angle method can be used for transmitter target location, especially of fixed radars. The proposed method belongs to the time-based localization techniques. The main advantage of this method is that it is able to locate targets that do not satisfy the condition of simultaneous irradiation of all the localization system receivers, which makes this method usable for surveillance of LPI/LPD non-cooperating targets.

In this paper, a model and computational algorithms of the method were described, and the target positioning accuracy was analyzed for constant angular velocity of the target antenna. The derivation of the method algorithm showed that the method is generally ambiguous in terms of determining the target location but that the false target location is a priori known. Next, it turned out that a limiting factor for practical application of the InA method was a very strict requirement for extraction accuracy of the receiving sensor irradiation time, as the standard deviation for the irradiation time measurement should not exceed 1 ms for sensors arranged with their mutual distances on the order of km. To reduce the effect of this limitation, an algorithm was proposed to improve the time of irradiation extraction.

Nevertheless, it should be noted that the accuracy of the InA method has not reached the accuracy of other time-based localization methods, such as the TDOA method. Therefore, this proposed method is considered a complementary method for other practical applications. A combination of TDOA/InA methods is a good example. Analysis of such combined methods will be the focus of our further work, as well as the extension of the InA method’s use for space sector scanning by the target antenna.

## Appendix A

The Jacobian matrix **J** is defined as:

(A1) $$J\left(\hat{T_{R}}\right)=\left[\frac{\partial f\left(\hat{T_{R}},S_{1..3}\right)}{\partial T_{R}}\right]=\left[\begin{array}{ccc} \frac{\partial x\left(\hat{T_{R}},\;S_{1..3}\right)}{\partial T_{R1}} & \frac{\partial x\left(\hat{T_{R}},\;S_{1..3}\right)}{\partial T_{R2}} & \frac{\partial x\left(\hat{T_{R}},\;S_{1..3}\right)}{\partial T_{R3}}\\ \frac{\partial y\left(\hat{T_{R}},\;S_{1..3}\right)}{\partial T_{R1}} & \frac{\partial y\left(\hat{T_{R}},\;S_{1..3}\right)}{\partial T_{R2}} & \frac{\partial y\left(\hat{T_{R}},\;S_{1..3}\right)}{\partial T_{R3}} \end{array}\right]$$

An example of a partial derivatives calculation is described below. For example, the *x_t_* target coordinate is obtained by equation

(A2) $$x_{t}=x_{t}^{*}.\cos\left(\gamma \right)-y_{t}^{*}.\sin\left(\gamma \right)+x_{o1}$$

where $x_{t}^{*}=\frac{c^{2}+r_{1}^{2}-r_{2}^{2}}{2.c}$ and $y_{t1,2}^{*}=\pm \sqrt{r_{1}^{2}-x_{t}^{*2}}$ are substitutes. Then, the partial derivate of *x_t_* with respect to *T_R1_* is

(A3) $$\frac{\partial x\left(\hat{T_{R}},\;S_{1..3}\right)}{\partial T_{R1}}=\frac{\partial x_{t}^{*}}{\partial T_{R1}}\cdot \cos\left(\gamma \right)-x_{t}^{*}\cdot \sin\left(\gamma \right)-\frac{\partial y_{t1,2}^{*}}{\partial T_{R1}}\cdot \sin\left(\gamma \right)-y_{t1,2}^{*}\cdot \cos\left(\gamma \right)+\frac{\partial x_{o1}}{\partial T_{R1}}$$

Next, the partial derivatives of substitutes $x_{t}^{*}$ and $y_{t1,2}^{*}$ with respect to *T_R_*_1_ are

(A4) $$\frac{\partial x_{t}^{*}}{\partial T_{R1}}=\frac{\left(2.c.\frac{\partial c}{\partial T_{R1}}+2.r_{1}.\frac{\partial r_{1}}{\partial T_{R1}}-2.r_{2}.\frac{\partial r_{2}}{\partial T_{R1}}\right).2.c-2.\left(c^{2}+r_{1}^{2}-r_{2}^{2}\right).\frac{\partial c}{\partial T_{R1}}}{4.c^{2}}$$

(A5) $$\frac{\partial x_{t}^{*}}{\partial T_{R1}}=\frac{\left(2.c.\frac{\partial c}{\partial T_{R1}}+2.r_{1}.\frac{\partial r_{1}}{\partial T_{R1}}-2.r_{2}.\frac{\partial r_{2}}{\partial T_{R1}}\right).2.c-2.\left(c^{2}+r_{1}^{2}-r_{2}^{2}\right).\frac{\partial c}{\partial T_{R1}}}{4.c^{2}}$$

The derivation continues in the same way until the partial derivatives of input variables, i.e., the inscribed angles, are found. There are

(A6) $$\frac{\partial \Delta \Phi_{1}}{\partial T_{R1}}=-\Omega$$

(A7) $$\frac{\partial \Delta \Phi_{1}}{\partial T_{R1}}=-\Omega$$

Finally, a complete partial derivative of *x_t_* with respect to *T_R_*_1_ can be calculated by inserting all of the above partial derivatives into Equation (A3). This approach takes into account that all the variables (or substitutes) used in the method algorithm can be depended on *T_R_*_1_. That means that it is necessary to calculate their partial derivatives with respect to *T_R_*_1_ too. The remaining partial derivatives of the Jacobian matrix can be derived in the same way.
